# Positively Charged Amino Acids in the Pestiviral E^rns^ Control Cell Entry, Endoribonuclease Activity and Innate Immune Evasion

**DOI:** 10.3390/v13081581

**Published:** 2021-08-10

**Authors:** Carmela Lussi, Elena de Martin, Matthias Schweizer

**Affiliations:** 1 Institute of Virology and Immunology (IVI), CH-3001 Bern, Switzerland; carmela_lussi@msn.com (C.L.); elena.demartin@vetsuisse.unibe.ch (E.d.M.); 2 Department of Infectious Diseases and Pathobiology, Vetsuisse Faculty, University of Bern, CH-3001 Bern, Switzerland; 3 Graduate School for Cellular and Biomedical Sciences (GCB), University of Bern, CH-3012 Bern, Switzerland

**Keywords:** pestivirus, bovine viral diarrhea virus (BVDV), viral endoribonuclease, IFN antagonist, immune evasion, glycosaminoglycan (GAG)-binding site, viral RNA, RNA-binding, site-directed mutagenesis, endocytosis

## Abstract

The genus *Pestivirus*, family *Flaviviridae*, includes four economically important viruses of livestock, i.e., bovine viral diarrhea virus-1 (BVDV-1) and -2 (BVDV-2), border disease virus (BDV) and classical swine fever virus (CSFV). E^rns^ and N^pro^, both expressed uniquely by pestiviruses, counteract the host’s innate immune defense by interfering with the induction of interferon (IFN) synthesis. The structural envelope protein E^rns^ also exists in a soluble form and, by its endoribonuclease activity, degrades immunostimulatory RNA prior to their activation of pattern recognition receptors. Here, we show that at least three out of four positively-charged residues in the C-terminal glycosaminoglycan (GAG)-binding site of BVDV-E^rns^ are required for efficient cell entry, and that a positively charged region more upstream is not involved in cell entry but rather in RNA-binding. Moreover, the C-terminal domain on its own determines intracellular targeting, as GFP fused to the C-terminal amino acids of E^rns^ was found at the same compartments as wt E^rns^. In summary, RNase activity and uptake into cells are both required for E^rns^ to act as an IFN antagonist, and the C-terminal amphipathic helix containing the GAG-binding site determines the efficiency of cell entry and its intracellular localization.

## 1. Introduction

Bovine viral diarrhea virus 1 (BVDV-1) and -2 (BVDV-2), border disease virus (BDV) and classical swine fever virus (CSFV) are positive-sense RNA viruses that were for a long time the only accepted species of the genus *Pestivirus* [1]. They represent important pathogens of livestock that cause large economical losses [2,3]. The isolation of numerous related species, starting with the description of a giraffe isolate in 1997 ([4] and references therein), to the more recent description of isolates from pigs, bats, harbor porpoise, or pangolins, amongst others [5,6,7,8,9,10,11,12], the Flaviviridae study group of the ICTV (International Committee on Taxonomy of Viruses) recently updated the classification, and renamed the species in a host-independent manner as “Pestivirus X” [13], with, e.g., the BVDV-1 and -2 viruses belonging to the species *Pestivirus A* and *B*, respectively.

A common feature of the pestiviruses is the presence of two proteins, N^pro^ and E^rns^. Both are reported to antagonize the host’s innate immune defense by blocking dsRNA-induced interferon (IFN) synthesis (summarized in [14]). While N^pro^ is a non-structural protein and is active in the cytoplasm of infected cells [15], E^rns^ is an envelope protein but is also secreted in a virus-free manner [16]. In addition, E^rns^ is a ribonuclease (RNase) with the proposed function to degrade the virus’s own single-(ss-) and double-stranded (ds) RNA to prevent activation of the host’s innate immune response [17,18,19,20]. For that purpose, soluble E^rns^ binds to glycosaminoglycans (GAGs) such as heparin or heparan sulfates present on the cell surface [21,22], followed by uptake via clathrin-mediated endocytosis [23], both prerequisites to efficiently block IFN synthesis.

Pestiviral E^rns^ contains two positive regions: ^139^KKGK^142^ and ^213^KKLENKSK^220^ (numbering, see Section 3.1). Both were examined for their heparin-binding potential but only mutants of the second region showed an effect on GAG-binding [24]. By contrast, an E^rns^ ΔC-term mutant that completely lacks the C-terminal GAG-binding site was still weakly active in inhibiting dsRNA-induced IFN expression in cell culture by measurement of Mx-expression [23]. This implemented that the positively charged region ^139^KKGK^142^ in addition to ^213^KKLENKSK^220^ could play a role in the binding of E^rns^ to GAGs.

In this study, we showed that extracellularly added, soluble E^rns^ is taken up into cultured cells into dot-like structures in the cytoplasm. Mutations affecting the C-terminal heparin-binding domain (residues 213–220) strongly reduced or eliminated cellular uptake, whereas mutations in the positive region (residues 139–142) more upstream did not affect cell entry of E^rns^ but rather reduced its RNase activity. Both properties, RNase activity and intracellular localization, are required for E^rns^ to inhibit the activation of the host’s innate immune response induced by immunostimulatory RNA. Finally, we demonstrated that the C-terminal domain of E^rns^ on its own determines the targeting to intracellular compartments, as GFP fused to the C-terminal amino acids of E^rns^ can be found concomitantly with wt E^rns^ in the same intracellular structures.

## 2. Materials and Methods

### 2.1. Cell Culture

Primary bovine turbinate (BT) cells were prepared at our institute from bovine fetuses that were obtained from a local abattoir. Eagles’ minimal essential medium (MEM) supplemented with 7% BVDV-free FCS, penicillin (100 IU/mL) and streptomycin (100 μg/mL) was used as cell culture medium. MEM was obtained from Biochrom (Bioswisstec AG, Schaffhausen, Switzerland) and FCS from PAA Laboratories (Lucerna-Chem AG, Lucerne, Switzerland). Cells were grown at 37 °C in a humidified 5% CO_2_ atmosphere.

### 2.2. Cloning of E^rns^ Mutants

Wild-type E^rns^ of the BVDV stain Ncp7 was subcloned into the pCI mammalian expression vector (Promega; kindly provided by P. Plattet) by Eurofins Genomics GmbH (Ebersberg, Germany). All mutants and GFP fusion constructs used in this study were subcloned using the “In-Fusion HD cloning kit” from Clontech (Takara Bio Europe SAS, Saint-Germain-en-Laye, France) as described for H30F and C171R [25]. Primers are available upon request. A mouse IgG kappa light chain signal sequence (ETDTLLLWVLLLWVPGSTG) for secretion and a twin-Strep-tag (SAWSHPQFEKGGGSGGGSGGSWSHPQFEK) are located in frame at the N-terminus and C-terminus, respectively. While the kappa sequence gets cleaved at the ER membrane upon protein expression, the strep-tag was retained at the C-term and used for purification and antibody detection.

### 2.3. E^rns^ Protein Expression and Purification

Expression and purification of Strep-tagged wt E^rns^ as well as of the mutants H30F and C171R were described in Lussi et al., 2018 [25]. All the other mutants used in this study were treated in a similar way. Briefly, plasmids were transfected into HEK293 cells in suspension at the “Protein Production and Structure Core Facility” (PTPSP) of the Swiss Federal Institute of Technology (EPFL) in Lausanne, Switzerland and after 7 days of cultivation, the cell supernatant containing the secreted E^rns^ proteins were purified using gravity flow Strep-Tactin Superflow columns (IBA GmbH, Göttingen, Germany). After reducing the pH to 7 by adding 1 M HCl, the concentration of all purified constructs was determined spectrophotometrically at 280 nm with a NanoDrop spectrometer (Thermo Fisher Scientific; Witec AG, Sursee, Switzerland).

### 2.4. Coomassie Staining

Purified E^rns^ proteins were analyzed under reducing or non-reducing conditions. Each construct (2 μg) was mixed with sample buffer (50 mM Tris buffer pH 6.8, 4% SDS, 12% glycerol and 0.01% Brilliant Blue G) either in presence or absence of the reducing agent 2% β-mercaptoethanol (2-ME). Reduced samples were boiled at 95 °C for 5 min. Proteins and a size marker (PageRuler™ Plus prestained protein ladder, Thermo Fisher Scientific, Reinach, Switzerland) were separated by sodium dodecyl sulfate-polyacrylamide gel electrophoresis (SDS-PAGE) as described below for Western blotting. Proteins were stained with Coomassie Brilliant Blue G250 (Bio-Rad Laboratories, Cressier, Switzerland) with first fixing for 30 min in 10% glacial acetic acid, 40% ethanol and followed by staining for 20 min in 0.1% Brilliant Blue G, 10% acetic acid and 45% ethanol. Prior to scanning, the gels were destained overnight in 10% glacial acetic acid, 20% ethanol.

### 2.5. RNase Activity Assay

A 300 bp dsRNA from the BVDV genome served as oligonucleotide substrate for the RNase activity assay. The double strand was achieved by incubating in vitro-transcribed ssRNA fragments of both, positive and negative polarity, from the 5′-UTR of the BVDV strain Ncp7 in boiling water followed by cooling down at room temperature as described [23]. Pre-dilution of the dsRNA was performed in 100 mM Tris/acetate buffer pH 5.5 and the E^rns^ constructs were diluted in elution buffer pH 7 (IBA GmbH). Equal volumes of substrate and E^rns^ were mixed together in a final volume of 10 µL. After incubation at 37 °C for one hour, 2× RNA loading dye (NEB; Bioconcept, Allschwil, Switzerland) was added and the digested RNA was separated on a 1% agarose gel containing ethidium bromide for 45 min at 100 V. Ethidium bromide staining was visualized by UV light using a U:Genius gel imaging system (Syngene; Labgene Scientific SA, Châtel-St-Denis, Switzerland).

### 2.6. Immunofluorescence (IF) Staining

BT cells were grown on cover glasses with thickness No. 1.5H (tol. ±5 μm) (Paul Marienfeld GmbH & Co. KG, Lauda-Königshofen, Germany) in a 24-well plate. After one day, cells were incubated with 10 ng/µL of Strep-tag purified wt or mutant E^rns^ for 30 min at 37 °C, diluted in MEM containing 2% FCS. After incubation, cells were washed twice with 0.1 mg/mL heparin sulfate to remove extracellularly bound E^rns^. Cells were incubated for 15 min with 4% formalin for fixation, followed by simultaneous permeabilization and blocking of unspecific binding sites by incubating the cells in PBS supplemented with 50 mM NH_4_Cl, 0.1% saponin and 2% BSA for 40 min. The same buffer was used for diluting the antibodies and for washing the cells. For the antibody treatment, the cover slide was headed upside down on a 40 μL drop of diluted antibody. A monoclonal mouse anti-E^rns^ antibody (50F4-10; kindly provided by T. Rümenapf, Institute of Virology, University of Veterinary Medicine, Vienna, Austria), a polyclonal rabbit eGFP Tag antibody (Thermo Fisher Scientific) and a monoclonal mouse anti-Strep-tag antibody (IBA GmbH) were used as primary antibodies at dilutions of 1/5, 1/200 and 1/100, respectively for 1.5 h. The binding of the primary antibodies was detected by fluorescently labeled donkey anti-mouse (H + L) Alexa 488, donkey anti-mouse Alexa 647 and donkey anti-rabbit Alexa 594 secondary antibodies (all from Jackson Immune Research; Milan Analytica AG, Rheinfelden, Switzerland) at a dilution of 1/400 for one hour. Finally, cells were washed with PBS and once with deionized water before mounting with ProLong Gold Antifade Mountant with DAPI (Life Technologies; Thermo Fisher Scientific). Slides were examined by 0.2 µm stacks over 10 µm with a 60× oil immersion objective (1.42 NA) using a DeltaVision Elite High-Resolution microscope (GE Healthcare Life Sciences). Pictures were deconvolved using the integrated softWoRx software and further processed using Fiji (ImageJ) [26] and Imaris software (RRID:SCR_007370; Bitplane, South Windsor, CT, USA).

### 2.7. Mx Assay

BT cells were seeded one day before they were either mock-treated or treated with one of the E^rns^ constructs for 30 min at 37 °C. E^rns^ was supplemented to the medium in a 1/5 dilution to yield a final concentration ranging from 5 to 0.04 ng/μL. Thereafter, the cells were washed two times with 0.1 mg/mL heparin sulfate prior to the addition of 2 μg/mL poly(IC). Then, 24 h later, the cells were directly lysed with M-PER™ mammalian protein extraction reagent (Pierce; Thermo Fisher Scientific) containing complete protease inhibitor cocktail (Roche; Sigma-Aldrich Chemie GmbH, Buchs, Switzerland). Lysed samples were mixed with SDS-PAGE sample buffer (50 mM Tris buffer pH 6.8, 4% SDS, 12% glycerol and 0.01% Brilliant Blue G) containing 2% 2-ME, boiled at 95 °C for 5 min and analyzed by Western blot.

### 2.8. Western Blot

Standard SDS-PAGE and Western blotting were performed using the equipment and protocols from Bio-Rad. Purified E^rns^ proteins or cell lysate from the Mx assay were loaded and separated on a 3% acrylamide stacking and 10% acrylamide separation gel. Electrophoresis in the stacking and separating gel were run at 80 and 120 V, respectively. Following electrophoresis, proteins were transferred to a nitrocellulose membrane (Amersham Biosciences) at 110 V at 4 °C for 90 min. The membranes were blocked for one hour at room temperature in PBS supplemented with 0.5% Tween-20 (PBS-T) and 5% low-fat milk powder. Target proteins were detected overnight at 4 °C with one of the following primary antibodies. For the Mx assay, a mouse monoclonal antibody against human MxA (kindly provided by Dr. J. Pavlovic from the Institute of Medical Virology, University of Zürich, Switzerland) was used as described [27], but with the Mx antibody being concentrated using Vivaspin filter (MWCO 10,000) and used at a dilution of 1/1000). For the visualization of the purified E^rns^ proteins, the same antibodies were used as described for immunostaining, i.e., the mouse anti-E^rns^ antibody and the mouse anti-Strep-tag antibody, at a dilution of 1/10 and 1/2500, respectively. As loading control for the individual lanes, a mouse anti-β-actin antibody (Sigma) was used at a dilution of 1/20,000. Peroxidase-conjugated donkey anti-mouse IgG (Jackson Immune Research), diluted 1/5000, was used as secondary antibody. Primary antibodies and secondary antibodies were diluted in PBS-T containing 0.5% low-fat milk powder. Protein expression was visualized using WesternBright ECL-HRP Substrate (advansta Inc.; Witec AG) as chemiluminescence substrate and recorded with a CCD camera (Fuji LAS-3000). Quantification of the signal intensities of each individual protein band was performed with the Advanced Image Data Analyzer AIDA software (raytest, Straubenhardt, Germany) and statistically analyzed with GraphPad Prism as described in the figure legends.

## 3. Results

### 3.1. Construction of E^rns^ Mutants

**Figure 1:**
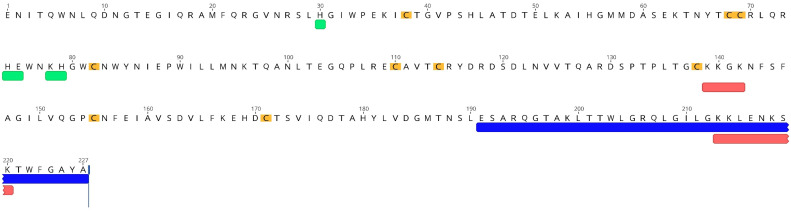
Amino acid sequence of wt E^rns^ of the BVDV strain Ncp7. Cysteine residues important for intra- and inter-molecular disulfide bridges (C171 for the latter) are highlighted in yellow. Residues reported to be part of the RNase active site are underlined in green, while the two conserved positively-charged regions are underlined in red (PR: ^139^KKGK^142^, and HBD: ^213^KKLENKSK^220^; compare Table 1). The last 37 amino acids at the C-terminus (which contain the amphipathic helix and the HBD) that were removed in the E^rns^-ΔC-term mutant are underlined in blue.

The full amino acid sequence of wild-type (wt) E^rns^ of the BVDV Ncp7 strain is given in Figure 1. RNase active site residues, cysteines responsible for intra- or intermolecular disulfide bridges and the potential heparin-binding domains are highlighted, as are the 37 amino acids at the C-terminus that are absent in the ΔC-term mutant.

To clearly identify the residues responsible for heparin-binding, various mutants of the two potential heparin-binding sites, ^139^KKGK^142^ (termed “positive region”, PR) and ^213^KKLENKSK^220^ (termed “heparin-binding domain, HBD; as only this region was previously reported to be responsible for heparin-binding [24]), were generated by substitution of positively charged lysines (K) with neutral alanines (A). In addition to the full mutants (^139^AAGA^142^ and ^213^AALENASA^220^) with all lysines changed, single or double site-mutants were constructed. To distinguish all these PR and HBD mutants, a code of 1 for wt lysine and 0 for alanine was used for each of the four lysine positions (see Table 1 for the complete list of PR and HBD mutants). In addition, two mutants lacking all seven lysine residues were generated, i.e., PR 000 + HBD 0000 and PR 000 + ΔC-term. As controls, an RNase inactive mutant (H30F) and a monomeric mutant (C171R) were used as described [19,25].

### 3.2. PR Mutations Result in the Formation of Higher Oligomers

**Figure 2:**
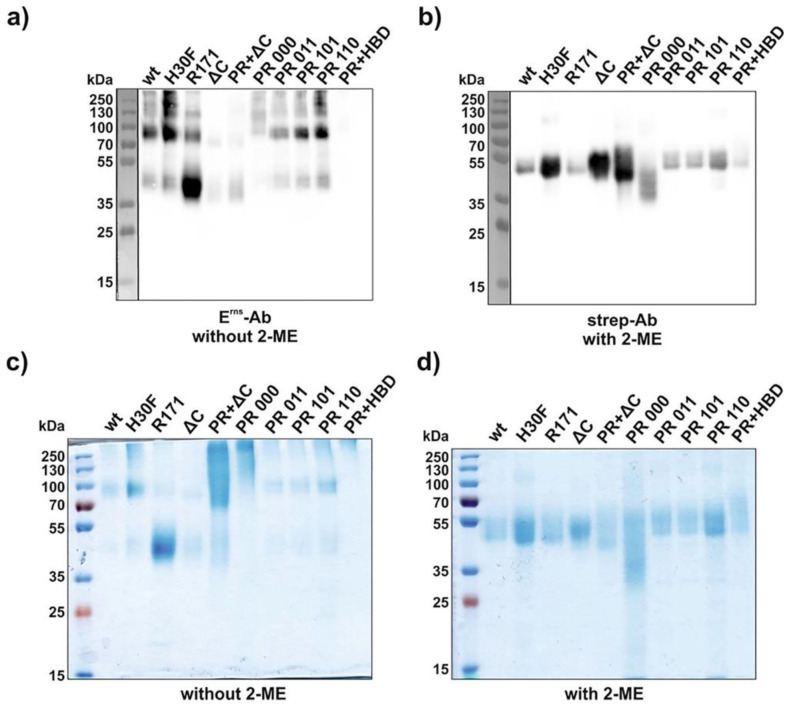
Molecular weight identification under reducing and non-reducing conditions. SDS-PAGEs were performed with various E^rns^ constructs (2 μg each, see Figure S1 for HBD mutants) and analyzed either by Western blot (**a**,**b**) or by Coomassie staining (**c**,**d**). Samples were incubated with or without β-mercaptoethanol (2-ME) as indicated in the figure. For Western blotting, an anti-Strep-tag and an anti-E^rns^ antibody were used under reducing (**a**) and non-reducing (**b**) conditions, respectively. The term “PR” and “HBD” in the lanes “PR + ΔC” and “PR + HBD” indicates the full mutants, i.e., PR 000 and HBD 0000. H30F represents the RNase-inactive mutant, and R171 represents the mutant C171R lacking the cysteine reported to be required for disulfide-linked E^rns^ dimerization. The size of the prestained protein ladder is indicated, with the ladder in panels a and b (separated by a vertical line) originating from an overlay of the chemiluminescence image of the antibody staining with a white light image capturing the prestained proteins of the ladder.

Soluble wt and mutant E^rns^ expressed by transiently transfected HEK cells were purified using a Strep-tag column as described in the Methods Section. The molecular weight of each construct was examined by conventional Western blot and by SDS-PAGE stained with Coomassie Brilliant Blue (Figure 2 and Figure S1). Both types of analysis were performed in the presence or absence of the reducing agent β-mercaptoethanol (2-ME) to verify the dimeric or monomeric state of the constructs. For Western blotting, however, the anti-E^rns^ antibody was active only under non-reducing conditions, whereas the anti-Strep-tag antibody was only functional at reducing conditions, as previously reported [25]. Under non-reducing conditions, the majority of the constructs were predominantly present at a size between 70 and 100 kDa except C171R, which appeared as a strong band at 40–50 kDa (Figure 2a,c and Figure S1a,c). This corresponds to a molecular weight of a dimeric (about 100 kDa) or monomeric (40–50 kDa) form of E^rns^. However, higher oligomeric structures than dimers were visible for wt and for most of the mutants. In the presence of 2-ME, all constructs lost their oligomeric/dimeric state and migrated as a single band at 40–50 kDa (Figure 2b,d and Figure S1b,d). Western blots under non-reducing conditions of all constructs containing a fully mutated PR (PR 000, PR 000 + ΔC, PR 000 + HBD 0000) were almost not detectable with the anti-E^rns^ antibody (Figure 2a). However, they were present as a smear in Coomassie staining ranging from 70 kDa up to 250 kDa under non-reducing conditions (Figure 2c).

### 3.3. Mutants of the PR Region of E^rns^ Are RNase Inactive

**Figure 3:**
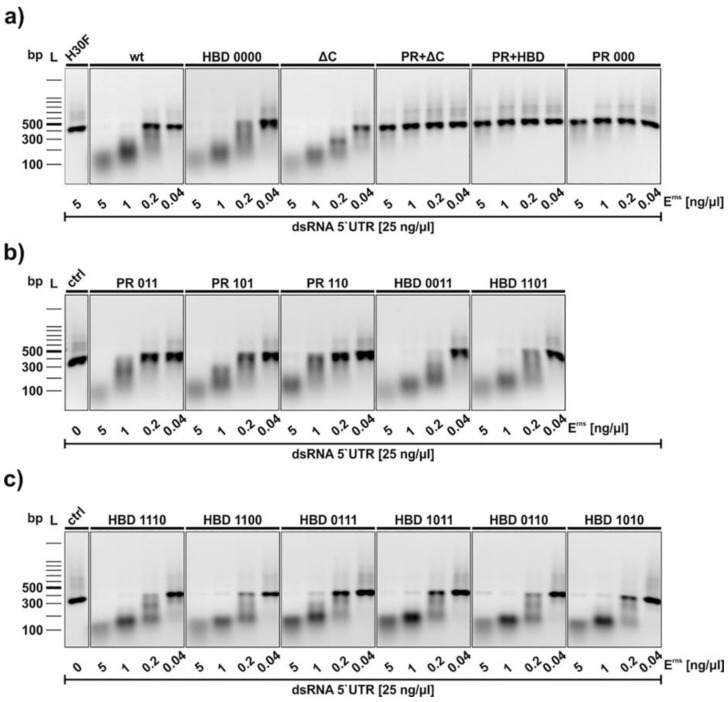
RNase activity assay of wt and mutant E^rns^. Constructs were incubated with an in vitro-transcribed 300 bp dsRNA of the 5′-UTR of the BVDV strain Ncp7. Panel (**a**) Analysis of wt E^rns^ and its full mutants. The terms PR and HBD in the lanes “PR + ΔC” and “PR + HBD” in this panel indicate the full mutants, i.e., PR 000 and HBD 0000. H30F represents the RNase-inactive mutant of E^rns^ used at the highest concentration, whereas the control (ctrl) in panels (**b**,**c**) indicates dsRNA incubated in the absence of any E^rns^ protein. Panels b and c contains PR- and HBD mutants with one or two of the lysines (code “1) replaced by alanine (code “0”). The concentrations of dsRNA and the proteins used are indicated. After one hour at 37 °C, the samples as well as a 100 bp DNA size marker were separated on a 1% agarose gel containing ethidium bromide. Bands were visualized by UV light. One representative experiment out of three is shown.

The functional activity of all E^rns^ constructs was examined with an RNase activity assay (Figure 3) with dsRNA as substrate [20,25]. E^rns^ wt and all mutants were incubated with a 300 bp dsRNA from the 5′-UTR of the BVDV strain Ncp7 for one hour and separated on a 1% agarose gel. Mutants with C-terminal modifications including all the HBD mutants as well as the ΔC-term mutant showed comparable degradation patterns as wt E^rns^. H30F, the RNase-inactive mutant, was used as a negative control (first line in Figure 3a). By contrast, the positive region full-mutant (PR 000), as well as the PR 000 + ΔC-term (PR + ΔC) and the PR 000 + HBD 0000 (PR + HBD) constructs, did not degrade dsRNA at all (Figure 3a). As all these mutants contain the PR 000 mutation, we determined which lysine residues were responsible for the RNase-inactive phenotype. RNase activity could be retained if only one of those lysines independent of the position was mutated to alanine (Figure 3b).

### 3.4. Lack of Entry of All E^rns^ Mutants with C-Terminal Modifications

**Figure 4:**
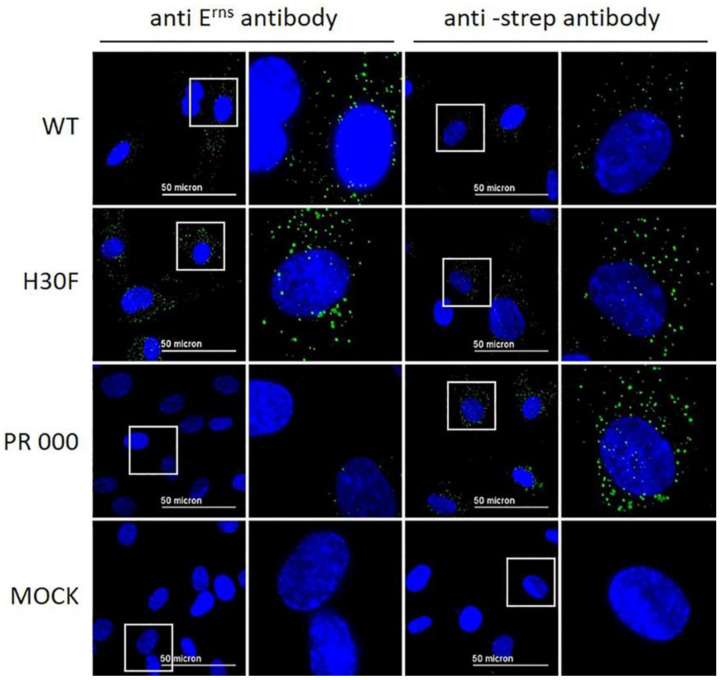
Wt E^rns^ and RNase-inactive mutants (H30F and PR 000) at a concentration of 10 ng/μL were incubated on BT cells for 30 min. E^rns^ was detected by immunofluorescence microscopy as described in the Methods Section using a primary antibody against E^rns^ (two columns on the left) or against the Strep-tag present at the C-terminus of each construct (two columns to the right) and a secondary antibody conjugated with Alexa 488 (green). Nuclei were stained with DAPI (blue) present in the mounting medium. The same image processing settings were used for mock and all constructs. For each picture, an area was selected (white square) for magnification and displayed to its right-hand side. One representative experiment out of three is shown.

**Figure 5:**
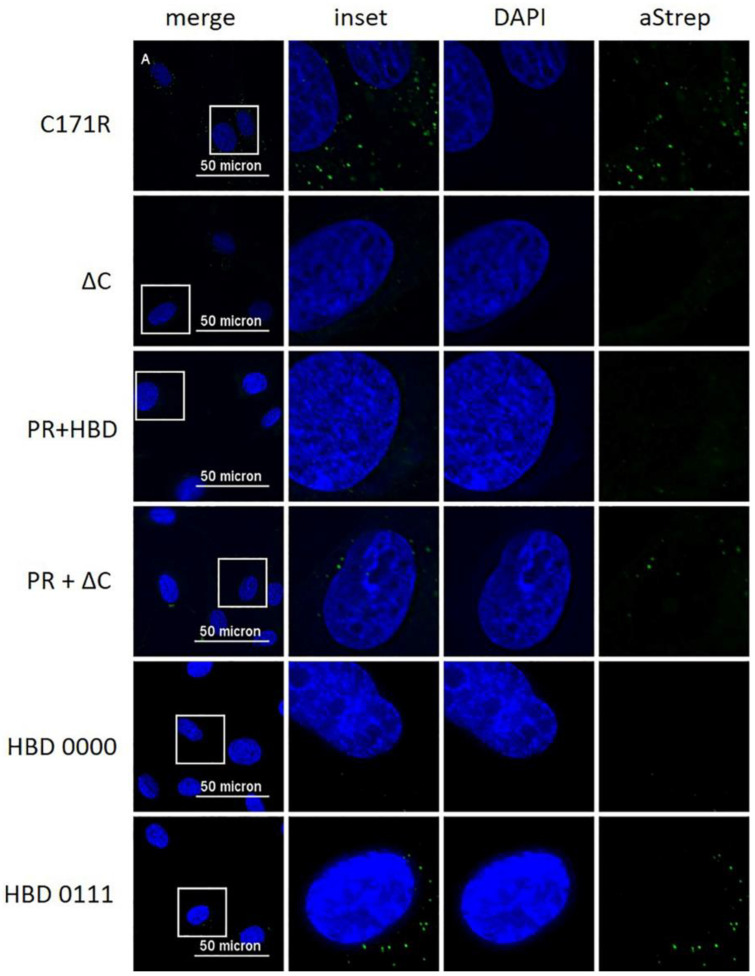
Immunofluorescence analysis of various mutants of E^rns^ with a Strep-tag antibody. Various E^rns^ mutants as indicated to the left at a concentration of 10 ng/μL were incubated on BT cells for 30 min. E^rns^ was detected by immunofluorescence microscopy as described in the Methods Section using a primary antibody (“aStrep”) against the Strep-tag present at the C-terminus of each construct and a secondary antibody conjugated with Alexa 488 (green). Nuclei were stained with DAPI (blue) present in the mounting medium. The same image processing settings were used for mock and all constructs. For each picture, an area was selected (white square) for magnification and displayed to its right-hand side (“inset” as merged image and DAPI and aStrep for the individual ones). One representative experiment out of three is shown.

Our previous results indicated that E^rns^ is required to enter the cells by endocytosis to block the activation of the host’s IFN response [23,27,28]. To provide further evidence for intracellular localization of E^rns^, we analyzed its uptake by immunofluorescence microscopy. To this end, bovine turbinate (BT) cells were treated with soluble E^rns^ and after 30 min of incubation, cells were stained for intracellular protein as described in the Methods Section. E^rns^ was detected by using either E^rns^- or Strep-tag-specific primary antibodies. Both, wt and H30F, appeared in a dot-like structure evenly distributed in BT cells detected with both primary antibodies (Figure 4), whereas the PR 000 mutant was visible in a similar pattern but only with the Strep-tag antibody (Figure 4), indicating that the epitope of the anti-E^rns^ antibody might be affected by mutations in the PR region. Therefore, all the other mutants were only tested with the Strep-tag-specific antibody (Figure 5). While the monomeric mutant as well as all PR mutants showed a wt-like pattern (Figure 4 and Figure S2), all constructs with a C-terminal modification (HBD 0000 and ΔC-term mutant) were not taken up by the cell. Single or double mutation within the HBD resulted in either less pronounced staining (HBD 0111 in Figure 5) or E^rns^ was not detected at all (Figure S2).

### 3.5. Both, RNase Activity and Endocytosis of E^rns^, Are Crucial for IFN Antagonism

**Figure 6:**
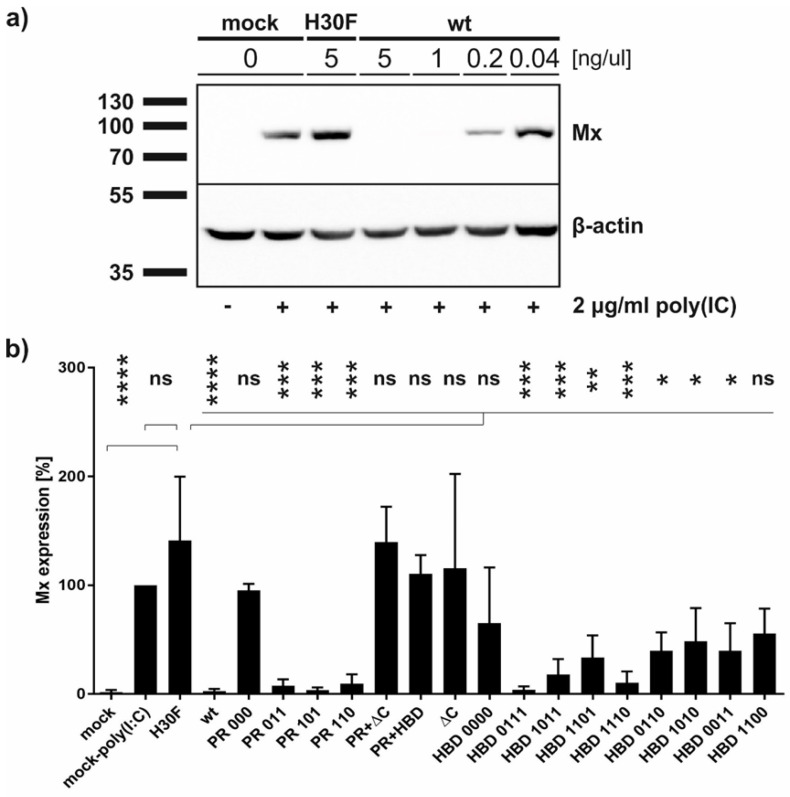
Ability of E^rns^ mutants to inhibit dsRNA-induced Mx expression. Cells were treated with E^rns^ for 30 min prior to the addition of poly(IC) as synthetic dsRNA. Cells were harvested 24 h later followed by Western blot analysis for the expression of Mx as indicator for the induction of IFN synthesis and for β-actin as loading control. The size of the proteins according to the protein ladder is indicated. Four independent replicates were performed for all constructs. (**a**) A representative Western blot of wt E^rns^ and the RNase-inactive mutant H30F at the concentrations as indicated is shown. (**b**) Quantification of the signal intensities of the Mx expression in Western blot as exemplified in panel a normalized to the corresponding signal of β-actin. The normalized signal of poly(IC)-induced Mx expression in the absence of E^rns^ (present on every gel) was set to 100%. Ordinary one-way analysis of variance (ANOVA) for multiple comparisons was performed, with the mean of each column being compared to the mean of the H30F column. Analysis was performed with the software GraphPad Prism version 7.03 and the significant differences of each construct at its highest concentration (5 ng/μL) are indicated with **** (*p* < 0.0001), *** (*p* < 0.001), ** (*p* < 0.01), * (*p* < 0.1), or ns (not significant).

It was shown that wt and monomeric E^rns^ are able to block poly(IC)-induced Mx expression [25]. In addition, a C-terminal truncated mutant of E^rns^ lacking the HBD region retained residual activity to inhibit dsRNA-induced IFN synthesis [23]. To investigate whether the various PR and HBD mutants are still acting as IFN antagonists at least in cell culture, we pre-treated BT cells for 30 min with each of the constructs followed by poly(IC) incubation for 24 h. Western blots of the cell extracts were performed and Mx expression was analyzed and quantified (Figure 6). In accordance with the RNase activity assay, all constructs containing PR 000 mutation were similarly unable to block poly(IC)-induced Mx expression, whereas single lysine mutations within this region showed significant Mx reduction. In contrast to our previous results using unpurified E^rns^ preparations [23], no significant activity was observed for the ΔC-term and HBD 0000 mutants, similar to the RNase-inactive mutant H30F. However, significant inhibition of Mx expression was observed for single and double mutants of HBD, with single mutants retaining three positively charged residues in the HBD region showing stronger inhibitory activity than the double mutants. The only exception to this was the mutant HBD 1100, which was not significantly different from the RNase-inactive mutant H30F. The significance of all concentrations tested for every mutant in comparison to the RNase-inactive H30F is summarized in Table S1.

### 3.6. The C-Terminus of E^rns^ Mediates Cellular Uptake of GFP

**Figure 7:**
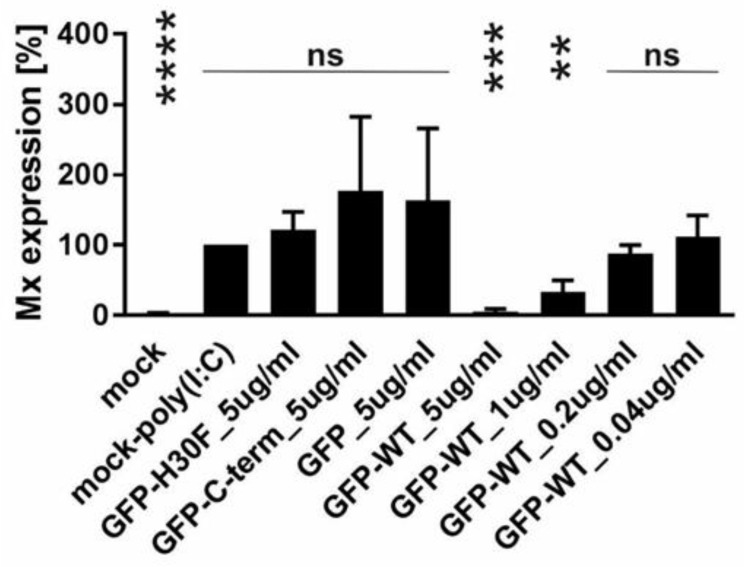
Ability of GFP-E^rns^ fusion proteins to inhibit dsRNA-induced Mx expression. Quantification of the signal intensities of poly(IC)-induced Mx expression was performed as described for Figure 6 with GFP fused to wt E^rns^ (GFP-wt) or to E^rns^-H30F (GFP-H30F), GFP fused to the C-terminal 37 amino acids of E^rns^ (GFP-C-term) and GFP alone at the concentrations as indicated. Statistical analysis was performed with the mean of four independent experiments of every sample compared to H30F at 5 ng/μL as described in Figure 6. **** (*p* < 0.0001), *** (*p* < 0.001), ** (*p* < 0.01), ns (not significant).

**Figure 8:**
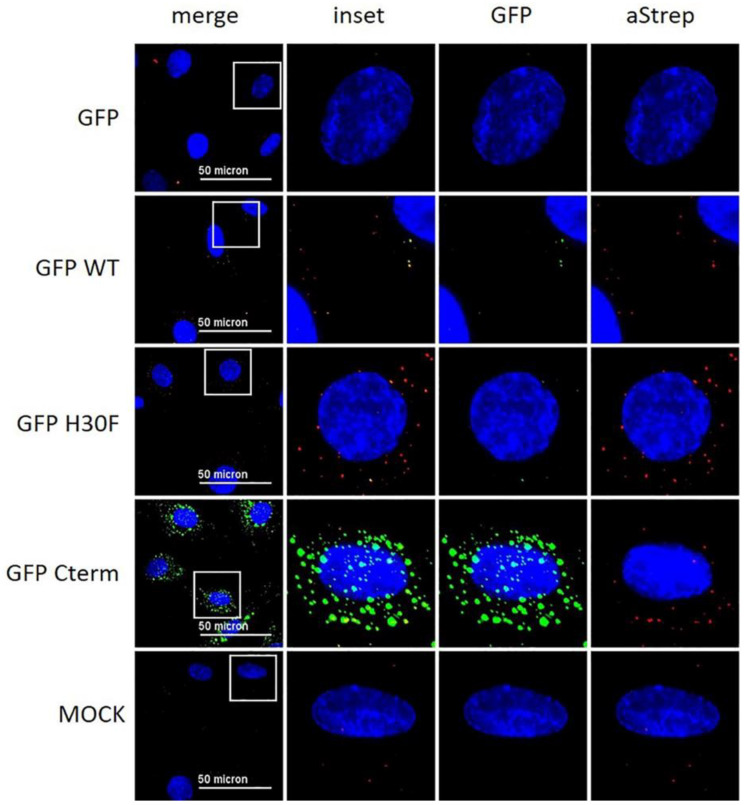
Immunofluorescence analysis of GFP-E^rns^ fusion proteins with anti-Strep-tag antibody. As indicated on the left side, GFP alone (“GFP”) and various GFP-E^rns^ fusion proteins (“GFP-WT”: GFP-wt E^rns^; “GFP H30F”: GFP-E^rns^-H30F; “GFP Cterm”: GFP-E^rns^-C-terminal end) at a concentration of 10 ng/μL were incubated on BT cells for 30 min. E^rns^ was detected by immunofluorescence microscopy as described in the Methods Section using a primary antibody against the Strep-tag present at the C-terminus of each construct and a secondary antibody conjugated with Alexa 647 fluorophore (red). The fluorescence signal of GFP (labeled on top) is shown in green. Nuclei were stained with DAPI (blue) present in the mounting medium. Adjustment of contrast and brightness was carried out with Fiji using the same setting for mock and all constructs. For each picture, an area was selected (white square), and the merged picture (inset) and each individual color are displayed magnified to the right-hand side. One representative experiment out of two is shown.

**Figure 9:**
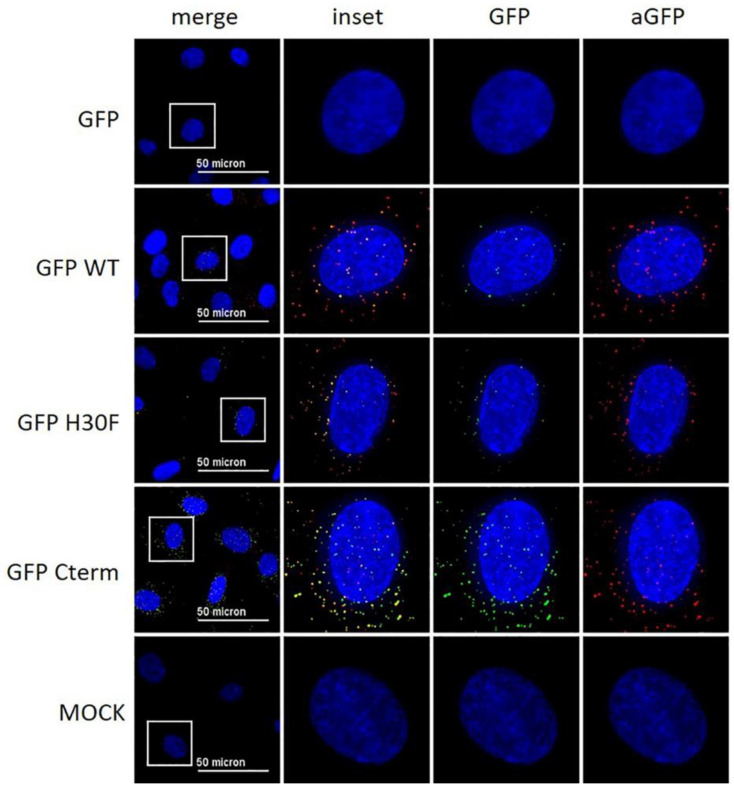
Immunofluorescence analysis of GFP-E^rns^ fusion proteins with an anti-GFP antibody. Various GFP-E^rns^ fusion proteins (“GFP-wt”: GFP-wt E^rns^; “GFP-H30F”: GFP-E^rns^-H30F; “ GFP Cterm”: GFP-E^rns^-C-terminal end), as well as GFP alone (“GFP”) at a concentration of 10 ng/μL, were incubated on BT cells for 30 min. The constructs were detected by immunofluorescence microscopy as described in the Methods Section using a primary antibody directed against GFP and a secondary antibody conjugated with an Alexa 594 fluorophore (red). The fluorescence signal of GFP (labeled on top) is shown in green. Nuclei were stained with DAPI (blue) present in the mounting medium. Adjustment of contrast and brightness was carried out with Fiji using the same setting for mock and all constructs. For each picture, an area was selected (white square), and the merged picture (inset) and each individual color are displayed magnified to the right-hand side. One representative experiment out of two is shown.

With the previous results, we demonstrated that the C-terminus, especially its heparin-binding domain HBD (^213^KKLENKSK^220^), is important for endocytosis of E^rns^. To provide further evidence for its role in the uptake of proteins into cells, we investigated the following fusion proteins for their uptake into cells and their ability to block dsRNA-induced IFN expression: GFP fused to the N-terminus of wt E^rns^ (separated by an (SGG)_3_ linker), GFP fused with the same linker to the RNase-inactive mutant E^rns^ H30F, and GFP fused to the C-terminal 37 amino acids of E^rns^ (i.e., the ones that were removed in the E^rns^-ΔC mutant) similarly separated by an (SGG)_3_ linker (termed GFP-C-term). Strep-tagged GFP without any addition was used as a control. GFP fused to E^rns^ exhibited equal RNase activity as wt E^rns^ alone (measured as described in Figure 3), whereas all the other GFP fusion constructs were inactive. Accordingly, only GFP fused to wt E^rns^ was able to significantly block poly(IC) induced Mx expression (Figure 7), whereas all the other chimeric proteins and GFP alone were not distinguishable from the RNase-inactive mutant H30F. Nevertheless, only GFP alone was not taken up into cells, whereas all chimeric constructs, i.e., GFP fused to wt- or H30F-E^rns^ or to the C-terminal end of E^rns^ comprising the HBD were visible as a dot-like structure within the cell (Figure 8 and Figure 9), similar to the ones observed for wt E^rns^ (Figure 4). These structures were similarly observed using either an antibody to the strep-tag (Figure 8) or one directly targeting GFP (Figure 9). No signal was observed with secondary antibody controls in the absence of any primary antibody (Figures S3 and S4). Whereas the antibody staining showed overall the same signal intensity for all the different fusion mutants, the GFP-C-term was often giving a saturated green fluorescence signal using the same settings for all fusion proteins.

To verify whether the C-term of E^rns^ on its own is sufficient to guide a protein such as GFP to similar intracellular structures as observed for wt E^rns^, we incubated GFP-C-term and wt E^rns^ simultaneously on the cells and analyzed their intracellular localization as described in Figure 8 and Figure 9. E^rns^ was visualized by the anti-E^rns^-antibody, whereas the GFP-C-term mutant was uncovered either with an anti-GFP antibody or by its GFP luminescence. A 3D reconstruction with Imaris was used to analyze the co-localization of the two proteins’ signals from every perspective. Some of the signals originating from wt E^rns^ and from the GFP-C-term mutant were clearly overlapping in all perspectives (Supplementary Video S1), confirming that both constructs were indeed localized at the same intracellular compartment.

## 4. Discussion

Previously, the positively charged region ^213^KKLENKSK^220^ (BVDV numbering according to Table 1) at the C-terminal end of E^rns^ was identified as the glycosaminoglycan (GAG)-binding site of BVDV E^rns^ (thus termed “heparin-binding domain HBD” in this study), whereas another region (^139^KKGK^142^) located more upstream was excluded as GAG-binding site (thus termed “positive region PR” in this study) based on its low affinity for heparin-binding [24]. By contrast, a ΔC-term mutant lacking the GAG-binding site (HBD region) showed residual activity as an IFN antagonist in a cell culture-based assay, indicating that the PR might partially retain its capacity of GAG-binding and enabling uptake of E^rns^ by clathrin-mediated endocytosis [23]. Here, we examined the importance of the positively charged amino acids at both sites for RNase activity in vitro, cellular uptake, and inhibition of dsRNA-induced IFN synthesis. For that purpose, all lysine residues in these regions were replaced by alanine, with the resulting mutants being named as positive region (PR 000) and heparin-binding domain (HBD 0000) mutants for AAGA and AALENASA, respectively. In addition, single- or double mutations within these sites as well as a ΔC-term mutant and various combinations thereof were constructed. Overall, our results indicate that the lysine residues in PR (positions 139-140 and 142) are rather involved in RNA- than in GAG-binding, and that at least three lysine residues within the second HBD motif ^213^KKLENKSK^220^ are required for endocytosis of E^rns^ and its activity as IFN antagonist. E^rns^ of CSFV contains an equivalent GAG-binding site, but GAG binding is increased by in vitro passaging of infectious virus due to the insertion of an additional positively charged amino acid at position 209 [21]. However, this increased binding to heparan sulfate was of minor importance in vivo [24,29,30]. In BVDV field isolates, by contrast, this position encoding for glycine is strongly conserved, and there is no selection for increased GAG-binding in vitro [24], except when passaging in cells lacking the receptor for E2 (CD46) [31]. Thus, there is good evidence that the first attachment of pestiviruses to its host cell might be due to the interaction of E^rns^ with glycosaminoglycans, which is followed by the engagement of a specific receptor for cell entry, but the precise role in vivo is still unknown [21,24,29,30,32].

Wt E^rns^ and the various mutants could all be expressed and affinity purified by the Strep-tag added at their C-terminal ends. Most of the proteins appeared as dimer under non-reducing conditions, with minor detection of monomeric and possibly oligomeric forms, as described earlier for BVDV and CSFV E^rns^ [25,33]. By contrast, the PR mutants (PR 000, PR 000 + ΔC, and PR 000 + HBD 0000) were only weakly detectable by Western blotting, especially with the anti-E^rns^ antibody at non-reduced conditions, and they rather appeared as a high molecular weight smear in Coomassie staining (Figure 2). The anti-E^rns^ antibody was also not able to stain the PR 000 mutants in the IF assay, but they were detectable with an anti-Strep-tag antibody (Figure 4 and Figure 5). Thus, we can deduce that the complete lack of positive charges in the PR region affects the conformation or the oligomerization status of E^rns^ that might lead to conformational changes or epitope covering that would explain the low binding affinity of the anti-E^rns^ antibody.

As reported previously [23], the ΔC mutant lacking the complete HBD fully retained its RNase activity, as did, therefore, all mutants with partial modification within the HBD. By contrast, all mutants harboring PR 000 were completely RNase-inactive despite the active site being outside this region. A change of only one out of three lysines in the PR retained the activity as endoribonuclease (Figure 3). According to the structure of the catalytic domain of E^rns^, the active site is mostly surrounded by positively charged patches, including the PR region [34], and it was suggested that this is important for binding of the RNA substrate [18,34]. Our results with alanine mutations support this assumption, despite the replacement of lysine by various amino acids might display a different phenotype. For example, the replacement of any lysine independent of the localization in the PR region retained RNase activity and the ability to block dsRNA-induced IFN expression in our study (Figure 6 and Table S1). However, K409A and K410T (corresponding to our alanine mutations PR 011 and PR 101, respectively) similarly retained RNase activity, but only K410T, not K409A, was able to block poly(IC)-induced Mx expression, whereas K412G (corresponding to PR 110) was RNase-inactive and thus unable to inhibit IFN synthesis induced by dsRNA [18]. Therefore, the specific role of each site-specific mutant in the conformation and possibly oligomerization of E^rns^ and its ability to act as an IFN antagonist remains to be determined.

We previously hypothesized that E^rns^ blocks the activation of the IFN response by degrading immunostimulatory ss- and dsRNA in an intracellular compartment, as (i) we could not detect any RNase activity of E^rns^ in cell culture medium alone [28], (ii) blocking of *extracellular* degradation of the dsRNA by complexation with an antimicrobial peptide (LL37) did not prevent E^rns^ to inhibit dsRNA-induced Mx expression [27], and (iii) prior endocytosis of E^rns^ was required to block IFN synthesis [23]. However, using crude, unpurified preparations of E^rns^ in cell culture supernatants, we were never able to provide evidence for its intracellular localization by immunofluorescence microscopy [23]. Using affinity-purified preparations of E^rns^, we were now able to visualize E^rns^ inside the cells by using a standard immunofluorescence protocol. After extracellular addition for 30 min, wt E^rns^, RNase-inactive mutant (H30F) and monomeric E^rns^ were all identified as cytoplasmic dot-like structures. Similar uptake into cells was previously shown for CSFV E^rns^ expressed by a baculovirus system in insect cells, but we could not confirm the localization to the nucleoli [35]. The kinetics of the uptake was similar, with uptake starting within minutes and being maximal at around 30 min [23,35], but we never observed any energy-independent uptake [35], but rather uptake via clathrin-mediated endocytosis [23]. The latter was completely prevented by the removal of the C-terminus (ΔC mutant), which is supported in this study by the complete lack of IF staining by this mutant (Figure 5). Similarly, we were unable to detect the mutant HBD 0000 inside the cells by immunofluorescence, providing further evidence that it is the GAG-binding site within the C-terminal amphipathic helix that determines attachment to and entry into cells. Accordingly, we were unable to intracellularly stain HBD mutants with one or more of the lysines changed into an alanine, except for the mutant HBD 0111 (Figure 5), and possibly HBD 1110 (Figure S2), where we could observe limited immunofluorescence staining.

Finally, the analysis of dsRNA-induced Mx expression (Figure 6 and Table S1) confirmed that the RNase activity E^rns^ and its ability to enter cells are both required for E^rns^ to act as an IFN antagonist. Accordingly, mutants with the complete exchange of all lysines in the PR and HBD (PR 000 and HBD 0000) and E^rns^-ΔC were unable to inhibit IFN synthesis, with PR 000 being able to enter the cells but being RNase-inactive, and vice versa for the HBD and E^rns^-ΔC mutants. The latter is in contrast to a previous study [18] where the mutant E^rns^ Δ483-497 (corresponding to Δ213–227 with the numbering used in this study) was able to inhibit dsRNA-induced Mx expression. The reason for this discrepancy is not known, but the incubation of insect cell-derived E^rns^ for 18 h, in contrast to only 30 min in our study, might have enabled partial, unspecific uptake of E^rns^, but this remains to be clarified. The results with the E^rns^-ΔC mutant are also in contrast to our previous study with E^rns^-ΔC exhibiting partial activity [23], but the use of purified protein preparations and the different mutants tested provide strong evidence that PR does not play a significant role in GAG-binding. Exchange of a single lysine in PR did not affect its activities, whereas the exchange of a single lysine, and even more pronounced by exchanging two lysines, in the HBD strongly reduced the capacity to inhibit poly(IC)-induced Mx synthesis, with the exception of HBD 0111 and HBD 1110. The fact that all the other HBD mutants, except these latter two, could not be detected by intracellular staining but still retained partial activity to block the activation of the innate immune system, might indicate that the Mx assay is more sensitive than immunostaining as only a few molecules of enzymatically active E^rns^ might be required to potently inhibit IFN induction [20]. By contrast, a larger number of molecules might be required to be detected by IF microscopy with the signal intensity being proportional to the number of proteins at a specific location.

To further confirm that the C-terminal part of E^rns^ including the HBD is sufficient for entry into the cells and that it determines its intracellular localization, we constructed various chimeric GFP mutants. GFP fused to wt E^rns^ or to its RNase-inactive mutant H30F was similarly taken up into cells as wt E^rns^ (Figure 8), and GFP-wt E^rns^, but not the chimeric protein with E^rns^-H30F, was still able to inhibit poly(IC)-induced Mx expression (Figure 7). This indicates that the fusion to GFP does not alter the activity of E^rns^ as an IFN antagonist and, therefore, does probably not influence its intracellular localization. Furthermore, GFP fused to only the C-terminal 37 amino acids of E^rns^ (i.e., the ones that are lacking in E^rns^-ΔC) was similarly taken up to cells and displayed dot-like structures in IF staining as wt E^rns^ (Figure 8 and Figure 9, Figure S3 and S4). The identity of these dot-like structures is not yet known, but the broad pH optimum of the RNase activity of E^rns^ with pH values from 4.5 to 7 [36] and the fact that E^rns^ is able to inhibit IFN induction by LL37-bound dsRNA that is released from LL37-complexation upon endosomal acidification [27], strongly suggest it to be endolysosomal-like structures. A 3D reconstruction of the IF images showed that at least some of the dots of wt-E^rns^ and of GFP fused to the C-terminal amino acids of E^rns^ (GPF-Cterm) co-localized, strongly indicating that the amphipathic helix including the HBD determines the intracellular localization of any protein fused to it. Again, these results partially contradict the translocation activity of the C-terminal domain reported previously [35], with the fusion peptides used at rather large concentrations, being found around the nucleus and concentrated in nucleoli, and being taken up also at 4 °C, i.e., in an energy-independent manner, which all could not be confirmed by us as discussed above. Nevertheless, the C-terminal domain of E^rns^ might provide a new tool to facilitate cellular uptake of a protein of interest and to target it to such endolysosomal compartments.

## 5. Conclusions

These results provide further evidence that E^rns^ requires its RNase activity and it needs to be taken up into the cells in order to act as a potent IFN antagonist. Mutations that affect either the RNase activity, e.g., by a mutation in the active site (H30F) or by reduced substrate binding (PR mutations), or that reduce the GAG-binding affinity of E^rns^ (HBD mutations or ΔC), strongly reduce the inhibition by E^rns^ of IFN expression induced by immunostimulatory RNA. Furthermore, the last 37 amino acids of the C-terminus of E^rns^, including the amphipathic helix and the GAG-binding domain, are sufficient for translocation of proteins such as GFP into the same cytoplasmic dot-like structures as wt E^rns^ is transported to. The exact nature of these structures remains to be determined but, based on the biochemical properties and the biological function of E^rns^, an endolysosomal localization can be envisaged. However, it has to be kept in mind that any mutation introduced might affect additional functions not studied here, such as binding to and retention in the lipid bilayer ([32] and references therein), processing by signal peptidase of the E^rns^-E1 junction in the viral polyprotein [37], or its participation in the assembly of infectious virus particles [38]. Thus, the C-terminal region of E^rns^ is a multifunctional domain [39] and further studies are required to disclose all its secrets.

## Figures and Tables

**Table 1:** Description of the E^rns^ mutants applied in the study. Various mutants of the two potential heparin-binding sites, ^139^KKGK^142^ (termed “positive region”, PR; numbering according to Figure 1) and ^213^KKLENKSK^220^ (termed “heparin-binding domain”, HBD), were generated by substitution of positively charged lysines (K) with neutral alanines (A). In addition to the full mutants (^139^AAGA^142^ and ^213^AALENASA^220^) with all lysines changed, single or double site-mutants were constructed. To distinguish all these PR-(left columns) and HBD-(right columns) mutants, a code of 1 for lysine and 0 for alanine was used in the names of each of the corresponding mutant (sites mutated are underlined in the sequence).

Name	Sequence	Name	Sequence
		HBD 0000	^213^AALENASA^220^
PR: wt E^rns^ 111	^139^KKGK^142^	HBD 0011	AALENKSK
HBD: wt E^rns^ 1111	^213^KKLENKSK^220^	HBD 1101	KKLENASK
		HBD 1110	KKLENKSA
		HBD 1100	KKLENASA
PR 000	^139^AAGA^142^	HBD 0111	AKLENKSK
PR 011	AKGK	HBD 1011	KALENKSK
PR 101	KAGK	HBD 0110	AKLENKSA
PR 110	KKGA	HBD 1010	KALENKSA

## Supplementary Materials

The following are available online at https://www.mdpi.com/article/10.3390/v13081581/s1, Figure S1: Molecular weight identification of E^rns^ mutants at reducing and non-reducing conditions; Figure S2: Intracellular localization of E^rns^ analyzed by immunofluorescence microscopy; Figure S3: Immunofluorescence analysis of GFP-E^rns^ fusion proteins (related to Figure 8): Figure S4: Immunofluorescence analysis of GFP-E^rns^ fusion proteins (related to Figure 9); Table S1: Statistical analysis of the ability of each mutant of E^rns^ to block poly(IC)-induced Mx expression in relation to the RNase-inactive mutant E^rns^-H30F; Video S1: Intracellular co-localization of wt E^rns^ and GFP-Cterm.
