# Supplementary material for: Positively Charged Amino Acids in the Pestiviral Erns Control Cell Entry, Endoribonuclease Activity and Innate Immune Evasion
Source: Viruses. 2021 Aug 10;13(8):1581. doi: 10.3390/v13081581 (PMC8402660; doi:10.3390/v13081581)
Supplement: Supplementary file 1 [file viruses-13-01581-s001.zip › Viruses_1301507-2021_R1_Lussi-et-al_Supplement.pdf]

viruses

Article

Positively charged amino acids in the pestiviral E^{rns} control cell entry, endoribonuclease activity and innate immune evasion

Carmela Lussi[1,2,3], Elena de Martin[1,2,3] and Matthias Schweizer[1,2,*]

[1] Institute of Virology and Immunology (IVI), CH-3001 Bern, Switzerland
[2] Department of Infectious Diseases and Pathobiology, Vetsuisse Faculty, University of Bern, CH-3001 Bern, Switzerland
[3] Graduate School for Cellular and Biomedical Sciences (GCB), University of Bern, CH-3012 Bern, Switzerland
[*] Correspondence: matthias.schweizer@vetsuisse.unibe.ch

Supplementary Materials:

The following supplementary materials are available online:

Table S1: Statistical analysis of the ability of each mutant of E^{rns} to block poly(IC)-induced Mx expression in relation to the RNase-inactive mutant E^{rns}-H30F.

Figure S1: Molecular weight identification of E^{rns} mutants at reducing and non-reducing conditions.

Figure S2: Intracellular localization of E^{rns} analysed by immunofluorescence microscopy.

Figure S3: Immunofluorescence analysis of GFP-E^{rns} fusion proteins (related to Figure 8).

Figure S4: Immunofluorescence analysis of GFP-E^{rns} fusion proteins (related to Figure 9).

Video S1: Intracellular co-localization of wt E^{rns} and GFP-Cterm.
3D analysis of IF images was performed to verify the co-localization of GFP fused to the C-term of E^{rns} (green) with wt E^{rns} (red) stained by an anti-E^{rns} antibody. The video focuses on showing from every perspective that a number of signals originating from the two proteins are overlapping (yellow), which indicates that both proteins localise in the same intracellular compartment.

E^{rns} mutant	5 ng/ul	1 ng/ul	0.2 ng/ul	0.04 ng/ul
wt	****	***	*	ns
PR 000	ns	ns	ns	ns
PR+ΔC	ns	ns	ns	ns
ΔC	ns	ns	ns	ns
PR 011	***	*	ns	ns
PR 101	***	***	**	ns
PR 110	***	*	ns	ns
PR+HBD	ns	ns	ns	ns
HBD 0000	ns	ns	ns	ns
HBD 0011	*	ns	ns	ns
HBD 1101	**	ns	ns	ns
HBD 1110	***	*	ns	ns
HBD 1100	ns	ns	ns	ns
HBD 0111	***	***	*	ns
HBD 1011	***	ns	ns	ns
HBD 0110	*	ns	ns	ns
HBD 1010	*	ns	ns	ns
GFP-E^{rns} wt	***	**	ns	ns
GFP-E^{rns} H30F	ns	ns	ns	ns
GFP-C-term	ns	n.d.	n.d.	n.d.
GFP	ns	n.d.	n.d.	n.d.

Table S1. Statistical analysis of the ability of each mutant of E^{rns} to block poly(IC)-induced Mx expression in relation to the RNase-inactive mutant E^{rns}-H30F. Analysis was performed as described for Figure 6, with the results highlighted in grey (5 ng/µl) already shown in Figure 6. ns: not significant; n.d.: not determined; **** ($p<0.0001$); ***($p<0.001$), ** ($p<0.01$), * ($p<0.1$).

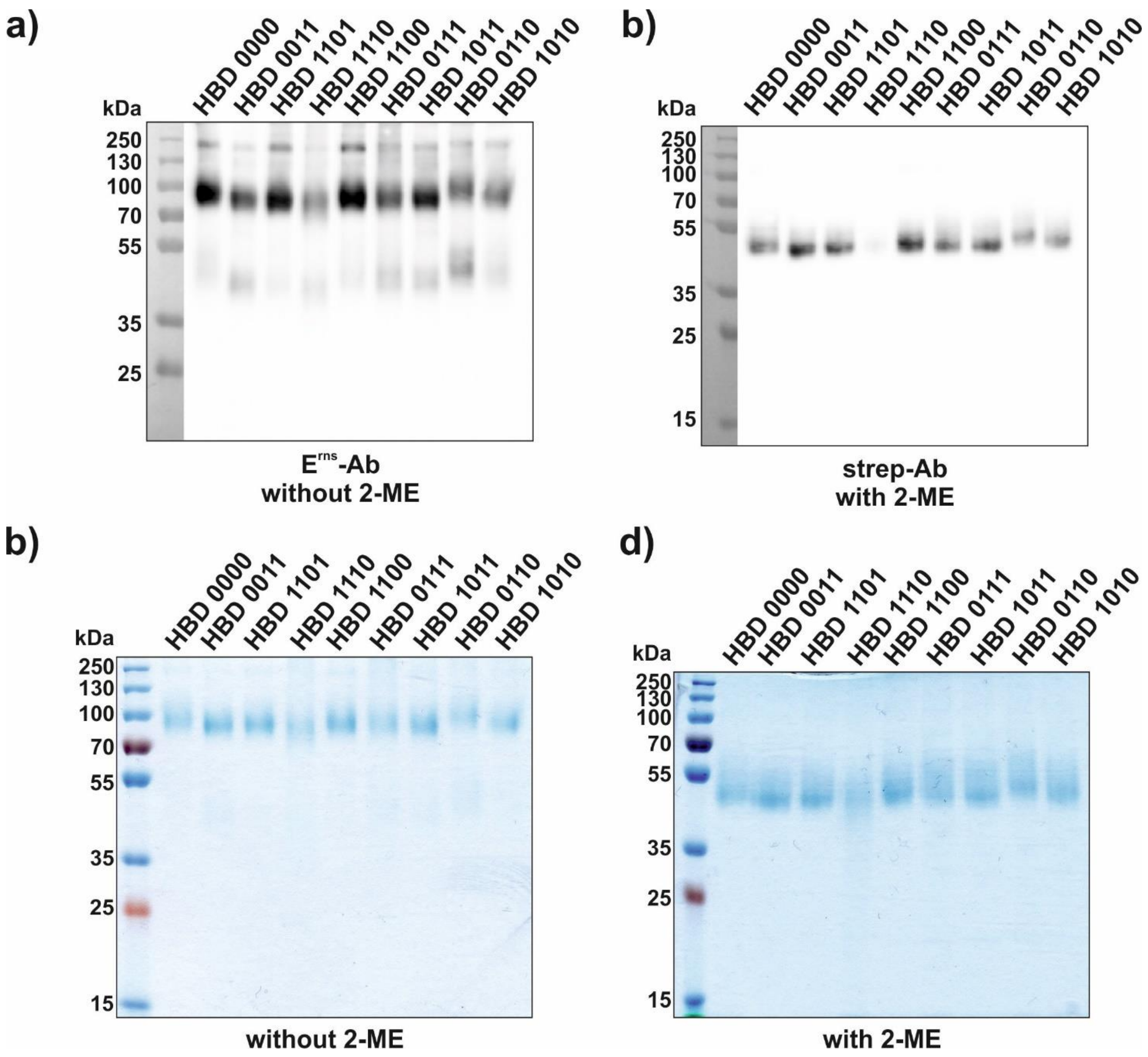

Figure S1. Molecular weight identification of E^rns mutants at reducing and non-reducing conditions.

SDS-PAGEs were performed with all HBD mutants of E^rns (2 μg each) and analysed either by western blot (a and b) or by Coomassie staining (c and d). Samples were incubated with or without β-mercaptoethanol (2-ME) as indicated in the figure. For western blotting, an anti-E^rns antibody (a) and an anti-Strep-tag antibody (b) was used under non-reducing (a) and reducing (b) conditions, respectively. PageRuler™ Plus Prestained Protein ladder was used for size determination. The size of the prestained protein ladder is indicated, with the ladder in panels a and b originating from an overlay of the chemiluminescence image of the antibody staining with a white light image capturing the prestained proteins of the ladder.

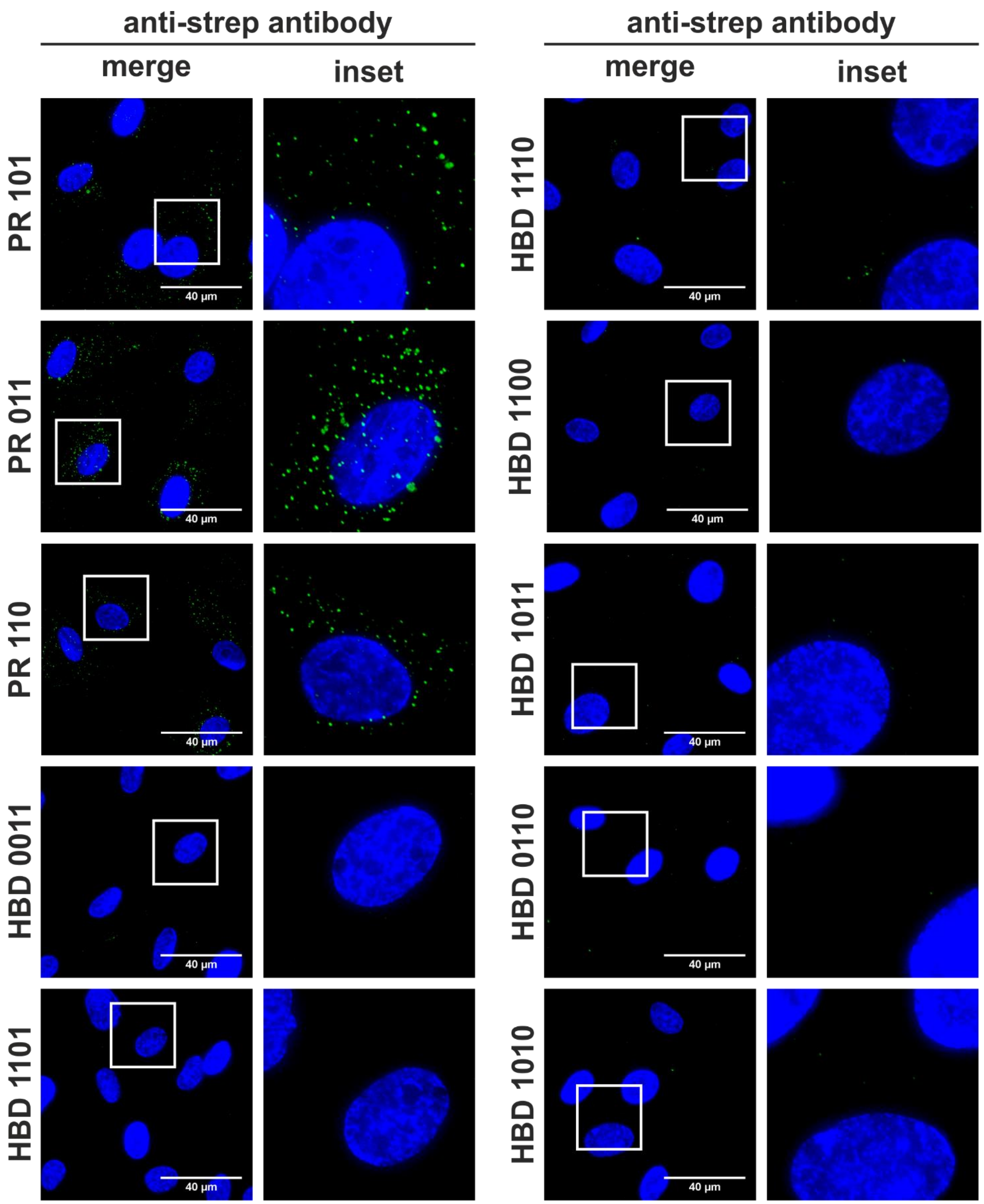

Figure S2. Intracellular localization of E^rns^ analysed by immunofluorescence microscopy.

PR and HBD mutants of E^rns^ with single or double lysine to alanine mutations at the corresponding regions (10 ng/µl) were incubated on BT cells for 30 minutes. Positions of mutated lysines are indicated in the name with the number "1" for lysine and "0" for alanine. Cells were fixed with 4% formalin, permeabilized with 0.1% saponin and blocked with 2% BSA. E^rns^ was detected with a primary antibody against a Strep-tag present at the C-terminus of each construct, and a secondary antibody conjugated with Alexa 488 (green). Nuclei were stained with DAPI (blue) present in the mounting medium. Adjustment of contrast and brightness was done with Fiji using the same setting used for Figure 4 and 5. For each picture, an area was selected (white square) for magnification and displayed to its right-hand side (inset). One representative experiment out of three is shown.

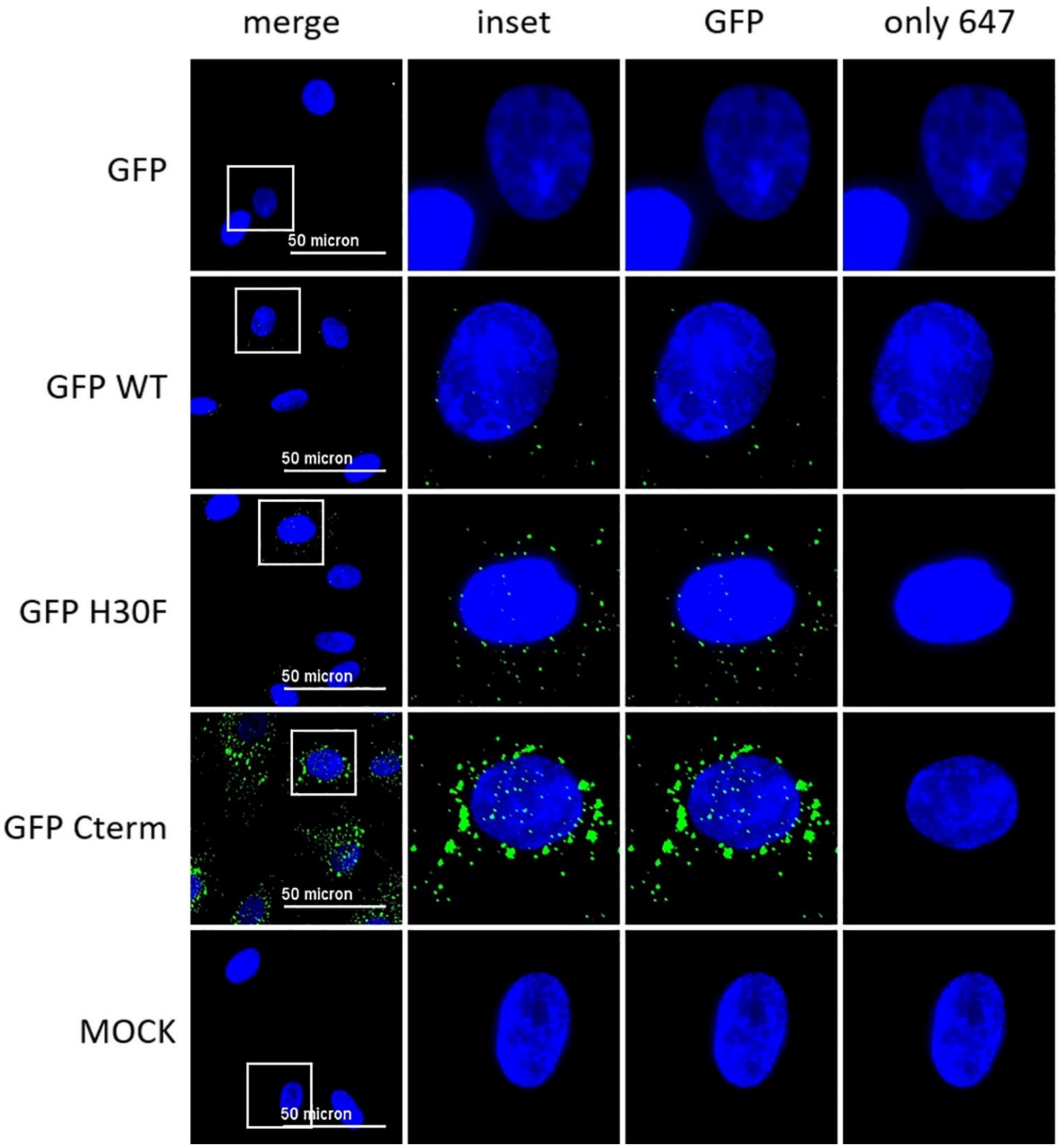

Figure S3. Immunofluorescence analysis of GFP-E^{rns} fusion proteins (related to Figure 8).

Various GFP-E^{rns} fusion proteins ("GFP WT": GFP-E^{rns} wt; "GFP H30F": GFP-E^{rns} H30F; "GFP Cterm": GFP-E^{rns} C-term) as well as GFP alone ("GFP") at a concentration of 10 ng/µl were incubated on BT cells for 30 minutes. Cells were fixed with 4% formalin, permeabilized with 0.1% saponin and blocked with 2% BSA. Fluorescence signal of GFP is shown in green. The last panels on the right-hand side represents the controls of the secondary antibody Alexa 647 alone in the absence of any primary antibody (compare Figure 8). Nuclei were stained with DAPI (blue) present in the mounting medium. Pictures were taken with a Delta Vision Elite high-resolution microscope combined with a deconvolution software. Adjustment of contrast and brightness was done with Fiji using the same setting as used for Figure 8. For each picture, an area was selected (white square) for magnification, and each individual color and the merged picture of the inset are displayed at the right-hand side. One representative experiment out of two is shown.

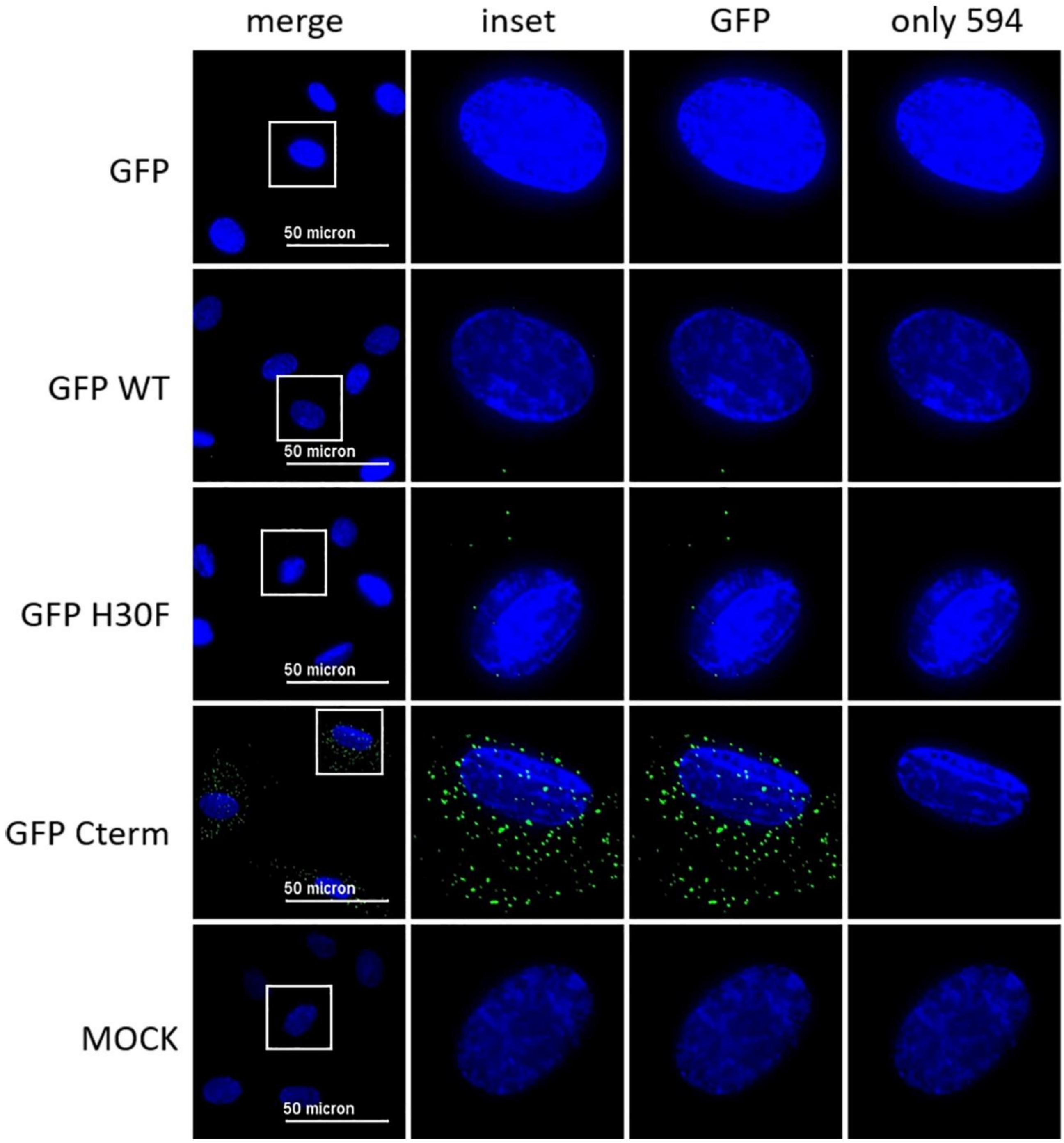

Figure S4. Immunofluorescence analysis of GFP-E^{rns} fusion proteins (related to Figure 9).

Various GFP-E^{rns} fusion proteins ("GFP WT": GFP-E^{rns} wt; "GFP H30F": GFP-E^{rns} H30F; "GFP Cterm": GFP-E^{rns} C-term) as well as GFP alone ("GFP") at a concentration of 10 ng/µl were incubated on BT cells for 30 minutes. Cells were fixed with 4% formalin, permeabilized with 0.1% saponin and blocked with 2% BSA. Fluorescence signal of GFP is shown in green. The last panels on the right-hand side represents the controls of the secondary antibody labeled with Alexa 594 alone in the absence of any primary antibody (compare Figure 9). Nuclei were stained with DAPI (blue) present in the mounting medium. Pictures were taken with a Delta Vision Elite high-resolution microscope combined with a deconvolution software. Adjustment of contrast and brightness was done with Fiji using the same setting as used for Figure 9. For each picture, an area was selected (white square) for magnification, and each individual color and the merged picture of the inset are displayed at the right-hand side. One representative experiment out of two is shown.
